# High Biofilm-Forming Ability and Clonal Dissemination among Colistin-Resistant *Escherichia coli* Isolates Recovered from Cows with Mastitis, Diarrheic Calves, and Chickens with Colibacillosis in Tunisia

**DOI:** 10.3390/life13020299

**Published:** 2023-01-20

**Authors:** Sana Dhaouadi, Amel Romdhani, Wafa Bouglita, Salsabil Chedli, Soufiene Chaari, Leila Soufi, Ameur Cherif, Wissem Mnif, Mohamed Salah Abbassi, Ramzi Boubaker Elandoulsi

**Affiliations:** 1 ISBST, BVBGR-LR11ES31, Biotechpole Sidi Thabet, University of Manouba, Ariana 2020, Tunisia; 2 Institut Supérieur de Biotechnologie de Sidi Thabet, Biotechpole Sidi Thabet, University of Manouba, Ariana 2020, Tunisia; 3 MEDIVET, Immeuble les Mimosas, 159 Avenue de l’UMA, La Soukra 2036, Tunisia; 4 Department of Chemistry, Faculty of Sciences and Arts in Balgarn, University of Bisha, P.O. Box 199, Bisha 61922, Saudi Arabia; 5 Institute of Veterinary Research of Tunisia, University of Tunis El Manar, Tunis 1006, Tunisia; 6 Laboratory of Bacteriological Research, Tunis 1006, Tunisia

**Keywords:** *E. coli*, colistin resistance, biofilm-forming ability, animal diseases, genetic relatedness

## Abstract

Background: *Escherichia coli* (*E. coli*) is one of the main etiological agents responsible for bovine mastitis (BM), neonatal calf diarrhea (NCD), and avian colibacillosis (AC). This study aimed to assess resistance and virulence genes content, biofilm-forming ability, phylogenetic groups, and genetic relatedness in *E. coli* isolates recovered from clinical cases of BM, NCD, and AC. Materials/Methods: A total of 120 samples including samples of milk (*n* = 70) and feces (*n* = 50) from cows with BM and calves with NCD, respectively, were collected from different farms in Northern Tunisia. Bacterial isolation and identification were performed. Then, *E. coli* isolates were examined by disk diffusion and broth microdilution method for their antimicrobial susceptibility and biofilm-forming ability. PCR was used to detect antimicrobial resistance genes (ARGs), virulence genes (VGs), phylogenetic groups, and Enterobacterial repetitive intergenic consensus PCR (ERIC-PCR) for their clonal relationship. Results: Among the 120 samples, 67 *E. coli* isolates (25 from BM, 22 from AC, and 20 from NCD) were collected. Overall, 83.6% of isolates were multidrug resistant. Thirty-six (53.73%) isolates were phenotypically colistin-resistant (CREC), 28.3% (19/67) were ESBL producers (ESBL-EC), and forty-nine (73.1%) formed biofilm. The *bla*_TEM_ gene was found in 73.7% (14/19) of isolates from the three diseases, whilst the *bla*_CTXM-g-1_ gene was detected in 47.3% (9/19) of isolates, all from AC. The most common VG was the *fim*A gene (26/36, 72.2%), followed by *aer* (12/36, 33.3%)*, cnf1* (6/36, 16.6%)*, pap*C (4/36, 11.1%), and *stx*1 and *stx*2 genes (2/36; 5.5% for each). Phylogenetic analysis showed that isolates belonged to three groups: A (20/36; 55.5%), B2 (7/36; 19.4%), and D (6/36; 16.6%). Molecular typing by ERIC-PCR showed high genetic diversity of CREC and ESBL *E. coli* isolates from the three animal diseases and gave evidence of their clonal dissemination within farms in Tunisia. Conclusion: The present study sheds new light on the biofilm-forming ability and clonality within CREC and ESBL-EC isolated from three different animal diseases in Tunisian farm animals.

## 1. Introduction

*Escherichia coli* (*E. coli*) is a highly diverse group of Gram-negative bacteria with the ability to colonize and persist in humans, warm-blooded animals, and abiotic environments [1,2]. However, some pathovars of *E. coli* are responsible for severe gastrointestinal diseases and a range of extra-intestinal infections in both humans and animals [3,4]. In addition, *E. coli* is one of the main etiological agents responsible for bovine mastitis (BM), neonatal calf diarrhea (NCD), and avian colibacillosis (AC), causing important economic losses [5,6,7].

There are two major groups of pathogenic *E. coli*: intestinal pathogenic *E. coli* (InPEC) and extraintestinal pathogenic *E. coli* (ExPEC). InPECs are divided into five main pathovars according to the clinical manifestation of the disease, the site of infection, and the virulence factors (VFs) repertoire. These pathovars include enterotoxigenic (ETEC), enteropathogenic (EPEC), enterohemorrhagic (EHEC), enteroinvasive (EIEC), and enteroaggregative (EAEC) [8]. Interestingly, ETEC and EPEC are also the most common pathovars associated with NCD [4,9]. On the other hand, ExPEC strains are classified into four pathovars, neonatal meningitis *E. coli* (NMEC), sepsis-associated *E. coli* (SEPEC), uropathogenic *E. coli* (UPEC), and avian pathogenic *E. coli* (APEC), based on the type of disorders they cause and their mode of interaction with the host [10]. APEC strains are the leading cause of avian colibacillosis responsible for diverse local and systemic infections in poultry, including chickens, turkeys, ducks, and many other avian species [7]. Although some bovine mastitis-associated *E. coli* (MAEC) strains carry genes associated with ExPEC virulence, most published data have not identified them as ExPEC due to their variable virulence factors (VFs) content. Indeed, the absence of a core set of VFs associated with bovine mastitis-associated *E. coli* has led to the proposal of MAEC as a distinct pathovar group [11]. Pathogenic *E. coli* are reservoirs of a wide range of VFs, including adhesins, invasins, toxins, and several uptake systems for various nutrients [12,13]. Additionally, it has been demonstrated that biofilms have important implications in the early stages of bacterial infection [4]. Indeed, the ability of pathogenic *E. coli* to adhere to epithelial cells, cause diseases, and enhance its antimicrobial resistance capacity is increased by biofilm formation [14].

Previous studies have revealed that APEC and ExPEC strains causing infections in humans are quite closely phylogenetically related and share some of the same virulence genes [15]. Moreover, EHEC strains have been involved in life-threatening gastrointestinal tract infections in humans, with bovines being their natural reservoir [16].

Apart from β-lactams, aminoglycosides, fluoroquinolones, and tetracyclines antibiotic families commonly used in animals [17]; colistin is considered one of the most critically important antimicrobials and has been increasingly used in animal husbandry [18]. Consequently, selection pressure exerted by inadequate use of antibiotics has led to the emergence of multidrug-resistant (MDR) *E. coli* (MDR), colistin-resistant *E. coli* (CREC), and Extended-spectrum β-lactamases *E. coli* (ESBL-EC).

The earlier reports so far from Tunisia have reported the occurrence of CREC and/or ESBL-EC from diseased [19,20,21] and healthy livestock [22,23]. However, it remains unclear if CREC and ESBL-EC isolates recovered from different animal diseases are genetically related and capable of biofilm formation. Therefore, this study aimed to (i) investigate the occurrence of CREC and ESBL-EC in cows with mastitis, diarrheic calves, and chickens with colibacillosis in Tunisia; (ii) assess their biofilm-forming ability and the molecular determinants of their resistance and virulence; and (iii) to determine their phylogenetic groups and their genetic relatedness.

## 2. Materials and Methods

### 2.1. Sampling and Sample Collection

In the period from February to April 2016, a total of 120 samples were collected from cows with mastitis and diarrheic calves in three adjacent farms (FIV, FV, and FVI) located in Bizerte and Ariana governorates in Northern Tunisia. Animals showed repetitive episodes of illness without death and were subjected to clinical examination by veterinarians. Following oral consent from animals’ owners, samples including mastitis milk and feces were collected from cows (*n* = 70) and calves (*n* = 50), respectively. Farms included in the present survey were characterized by a number of animals ranging from 15 to 32 and were not under control by official veterinary services. According to animals’ owners, the most commonly used antibiotics in treating diseased animals in these farms included β-lactams, aminoglycosides, fluoroquinolones, and tetracyclines. Cows and calves included in the present investigation shared the grazing environment, food, and water sources. In addition, cow’s milk was used to feed young calves. All samples were transported to the laboratory within a few hours of collection in refrigerated boxes and processed immediately. Twenty-two *E. coli* isolates previously identified from chickens who died of colibacillosis in three different farms (I, II and III) located in Nabeul, Ben Arous, and Zaghouane governorates in Northeast Tunisia were included in the present study for further analysis [20].

### 2.2. Isolation and Bacteria Identification

One hundred microliters from each sample was placed in brain heart infusion broth (Oxoid Ltd., Basingstoke, UK) and incubated aerobically at 37 °C for 24 h. Then, 10 µL of culture suspensions were seeded onto MacConkey agar (Merck, Darmstadt, Germany) plates and incubated overnight at 37 °C. Isolates with typical *E. coli* morphology were selected and seeded onto Endo agar (Merck) and incubated overnight at 37 °C. One presumptive colony per sample was selected and identified by conventional methods including Gram staining and biochemical tests (oxidase, catalase, urea-Indole, lactose, and glucose fermentation gas production ability in Kigler-Hajna agar) and by an API 20E system (BioMerieux, Marcy l’Etoile, France). Bacterial DNA for polymerase chain reaction (PCR) analysis was prepared by boiling a loopful of bacteria in 400 µL of TE buffer (10 mMTris–2 mM EDTA) for 10 min, followed by centrifugation for 15 min at 10,000× *g*. Subsequently, isolates were confirmed as *E. coli* using species-specific PCR targeting the *uid*A gene encoding for β-glucuronidase structural protein [24] (Table 1).

### 2.3. Antimicrobial Susceptibility Testing and Screening for ESBL Production

Antimicrobial susceptibility of all *E. coli* isolates was determined using the disc diffusion method and interpreted according to the Clinical and Laboratory Standards Institute [25] and the European Committee on Antimicrobial Susceptibility Testing guidelines [26]. The following antibiotics (Oxoid) were used (µg/disk): Ampicillin (AMP, 10 µg), nalidixic acid (NA, 30 µg), tetracycline (TET, 30 µg), trimethoprim–sulfamethoxazole (SXT, 1.25/23.75 µg), streptomycin (STR, 10 µg), cefotaxime (CTX, 30 µg), cefoxitin (FOX, 30 µg), ceftiofur (XNL, 30 µg), cefsulodine (CFZ, 30 µg), gentamicin (GN, 30 µg), enrofloxacin (ENR, 5 µg), imipenem (IMP, 30 µg), meropenem (MEM, 30 µg), ertapenem (ETP, 30 µg), chloramphenicol (CHL, 30 µg), and colistin (CST, 50 µg). The double-disk synergy test (DDST) with cefotaxime (CTX, 30 µg), ceftazidime (CAZ, 30 µg), aztreonam (ATM, 30 µg), and cefepime (FEP, 30 µg) in the proximity of amoxicillin-clavulanic acid (AMC, 20/10 µg) was used for the screening of ESBL production. *E. coli* ATCC25922 and *Klebsiella pneumonia* ATCC700603 were used as ESBL-negative and positive control strains, respectively. The isolates were defined as multidrug-resistant (MDR) if they exhibited resistance to at least one agent belonging to three or more antimicrobial families [27].

### 2.4. Colistin Susceptibility Testing and Screening of Colistin Resistance Genes

The minimum inhibitory concentration (MIC) of colistin was determined using the broth microdilution method (BMD) according to the CLSI guidelines [28]. Dilution methods were performed using colistin sulfate (Sigma-Aldrich, Merck, Darmstadt, Germany) tested over a range from 0.25 to 128 µg/mL. All experiments were performed in triplicate. *E. coli* ATCC 25922 was used as a quality control strain. The *mcr*-1, *mcr*-2, *mcr*-3, and *mcr*-4 genes encoding for colistin resistance were investigated by PCR in all isolates with MIC ≥2 µg/mL as described elsewhere [18,29,30,31].

### 2.5. Detection of Resistance Genes in CREC Isolates

The 36 CREC isolates (12 from avian colibacillosis, 18 from mastitis, and 6 from diarrhea) were selected for further molecular characterization. CREC isolates were screened by PCR for the presence of antimicrobial resistance genes conferring resistance to streptomycin (*aad*A, *str*A, *str*B), phenicols (*cml*A, *flo*R), tetracyclines (*tet*A, *tet*B), trimethoprim (*dfr*AI, *dfr*VII), and sulfonamides (*sul*1, *sul*2) as previously described [32,33] (Table 1).

### 2.6. Detection of β-Lactamase-Encoding Genes

All ESBL-EC isolates were screened for the presence of five β-lactamase-encoding genes (*bla*_TEM_, *bla*_SHV_, *bla*_CTX-M-g-1_, *bla*_CTX-M-g-8_, and *bla*_CTX-M-g-9_) using PCR conditions as previously described [24,32] (Table 1).

### 2.7. Biofilm Formation Assay

The biofilm formation ability of the 67 *E. coli* isolates was performed in 96-well microtiter plates [34]. Briefly, an overnight culture was diluted (1:100) in TSB containing 1% glucose and inoculated onto microtiter plates at 37 °C for 18 h without aeration. The free-floating planktonic bacteria were removed and washed, dried for 60 min at 60 °C, and stained with 0.06% crystal violet. The biofilm was quantified in duplicate, after adding 200 μL of 95% ethanol using a microtiter plate reader by an enzyme-linked immunosorbent assay plate reader at 570 nm (BioRad). Each strain was tested in triplicate and each assay was performed in duplicate. *E. coli* ATCC25922 and *S. epidermidis* strain ATCC12228 were used as positive and negative controls, respectively. The isolates were classified as strong biofilm producer: 4 × OD_C_< OD; moderate biofilm producer: 2 × ODc < OD ≤ 4 × ODc; weak biofilm producer: ODc < OD < 2 × ODc; and no biofilm producer: OD ≤ ODc [35]. The cut-off value (ODc) is defined as three standard deviations (SD) above the mean OD of the negative control (TSB plus 1% glucose, without bacterial cells) [35].

### 2.8. Detection of Virulence and Biofilm Encoding Genes

Biofilm and virulence-associated genes (*fim*A, *pap*C, *hly*, *aer*, *cnf*1, *stx*1 and *stx*2) were investigated in CREC isolates by PCR using sets of primers as described in previous studies [36,37] (Table 1).

### 2.9. E. coli Phylogenetic Typing

Phylogenetic groups (A, B_1_, B_2_, or D) and sub-groups (A_0_, A_1_, B_1_, B2_2_, B2_3_, D_1_, and D_2_) of all *E. coli* isolates were determined using a triplex PCR targeting the *chu*A, *yja*A genes, and the DNA fragment *tsp*E4.C2 as described by Clermont et al., 2000 [38] and Escobar-Paramo et al. (2006) [39] (Table 1).

**Table 1:** Primers, amplicon size and annealing temperature used for the detection of resistance genes, integrons, virulence genes, phylogenetic groups, and genotyping of *E. coli* isolates.

Primer Name	Oligonucleotide Sequence (5′-3′)	Amplicon Size (bp)	Annealing Temp. °C	Specificity	Reference
***E. coli* Identification**					
*Uid*A	F: ATCACCGTGGTGACGCATGTCGC	486	51	β-glucuronidase enzyme	[24]
	R: CACCACGATGCCATGTTCATCTGC				
**Resistance Genes**					
*mcr*1	F: CGGTCAGTCCGTTTGTTC	309	58	Colistin	[18]
	R: CTTGGTCGGTCTGTAGGG				
*mcr*2	F:TGTTGCTTGTGCCGATTGGA	567	58		[29]
	R: AGATGGTATTGTTGGTTGCTG				
*mcr*-3	F: TTGGCACTGTATTTTGCATTT	542	50		[30]
	R: TTAACGAAATTGGCTGGAACA				
*mcr*-4	F: ATTGGGATAGTCGCCTTTTT	487	56		[31]
	R: TTACAGCCAGAATCATTATCA				
*bla*_TEM_	F: ATTCTTGAAGACGAAAGGGC	1150	60	Bêtalactamases	[32]
	R: ACGCTCAGTGGAACGAAAAC				
*tet*(A)	F:AATTCTGAGCACTGTCGC	937	62	Tetracyclines	
	R: CTGCCTGGACAACATTGCTT				
*tet*(B)	F: CTCAGTATTCCAAGCCTTTG	416	57		
	R: CTAAGCACTTGTCTCCTGTT				
*str*A	F: ATTCTGACTGGTTGCCTGTC	1562	55	Streptomycin	
	R: CGCAGATAGAAGGCAAGG				
*str*B	F: TTCTCATTGCGGACAACCT	1562	55		
	R: TAGATCGCGTTGCTCCTCTT				
*Dfr*AI	F: GTGAAACTATCACTAATGG	474	55	Trimethoprim	
	R: TTAACCCTTTTGCCAGATTT				
*Dfr*VII	F: TTGAAAATTTCATTGATT	474	55		
	R: TTAGCCTTTTTTCCAAATCT				
*sul*1	F:TGGTGACGGTGTTCGGCATTC	789	63	Sulfamides	
	R: GCGAGGGTTTCCGAGAAGGTG				
*sul*2	F: CGGCATCGTCAACATAACC	722	50		
	R: GTGTGCGGATGAAGTCAG				
*aad*A	F: GCAGCGCAATGACATTCTTG	282	60	Streptomycin	[33]
	R: ATCCTTCGGCGCGATTTTG				
*flo*R	F: CACGTTGAGCCTCTATAT	868	55	Florfenicol	[32]
	R: ATGCAGAAGTAGAACGCG				
*cmlA*	F: TGTCATTTACGGCATACTCG	455	55	Chloramphenicol	
	R: ATCAGGCATCCCATTCCCAT				
*bla*_SHV_	F: CACTCAAGGATGTATTGTG	885	52	β-lactamases	[24]
	R: TTAGCGTTGCCAGTGCTCG				
*bla*_CTX-M-g-1_	F: GTTACAATGTGTGAGAAGCAG	1041	50		
	R: CCGTTTCCGCTATTACAAAC				
*bla*_CTX-M-g-8_	F: TGATGAGACATCGCGTTAAG	666	52		
	R: TAACCGTCGGTGACGATTTT				
*bla*_CTX-M-g-9_	F: GTGACAAAGAGAGTGCAACGG	856	62		
	R: ATGATTCTCGCCGCTGAAGCC				
**Virulence Genes**					
*fim*A	F: GTTGTTCTGTCGGCTCTGTC	447	55	Type 1 Fimbriae	[36]
	R: ATGGTGTTGGTTCCGTTATTC				
*aer*	F: TACCGGATTGTCATATGCAGACCGT	602	55	Aerobactin iron uptake system	
	R: AATATCTTCCTCCAGTCCGGAGAAG				
*stx*1	F: CTGGATTTAATGTCGCATAGTG	150	55	Type 1 Shiga-toxin	[37]
	R: AGAACGCCCACTGAGATCATC				
*stx*2	F: GGCACTGTCTGAAACTGCTCC	255	55	Type 2 Shiga-toxin	
	R: TCGCCAGTTATCTGACATTCTG				
*hlyA*	F: AACAAGGATAAGCACTGTTCTGGCT	1177	55	Alpha-hemolysin	[36]
	R: ACCATATAAGCGGTCATTCCCGTCA				
*cnf*1	F: AAGATGGAGTTTCCTATGCAGGAG	498	55	Cytotoxic necrotizing factor 1	
	R: CATTCAGAGTCCTGCCCTCATTATT				
*pap*C	F:GACGGCTGTACTGCAGGGTGTGGCG	328	55	P Fimbriae	
	R: ATATCCTTTCTGCAGGGATGCAATA				
**Phylogenetic Groups**					
*chu*A	F: GACGAACCAACGGTCAGGAT	279	55	Phylogenetic groups	[38,39]
	R: TGCCGCCAGTACCAAAGACA				
*yja*A	F: TGAAGTGTCAGGAGACGCTG	211	55		
	R: ATGGAGAATGCGTTCCTCAAC				
*tsp*E4.C2	F: GAGTAATGTCGGGGCATTCA	152	55		
	R: CGCGCCAACAAAGTATTACG				
**Genotyping**					
ERIC	F: ATGTAAGCTCCTGGGGATTCAC	*	52	Enterobacterial Repetitive Intergenic Consensus	[40]
	R: AAGTAAGTGACTGGGGTGAGCG				

* Bands profile.

### 2.10. E.coli Molecular Typing by ERIC-PCR

CREC isolates were fingerprinted by ERIC-PCR as described by Bilung et al. (2018) [40] (Table 1) and different ERIC-PCR profiles were analyzed visually and numerically according to Tenover et al. (1998) [41]. Then, the phylogenetic tree was established using MVSP 3.2 software. The comparison between ERIC-PCR profiles was conducted using the Jaccard coefficient, and a dendrogram was constructed using the unweighted pair group method with arithmetic mean (UPGMA).

### 2.11. Statistical Analysis

Statistical analysis was performed in IBM SPSS 22.0. A chi-squared test χ² using the Pearson Chi-square test was employed to estimate differences between colistin resistance, virulence genes, biofilm formation, and ESBL production rates in *E. coli* from the three animal diseases, whereby a probability of less than 0.05 was considered statistically significant.

## 3. Results

### 3.1. Collected E. coli Isolates

A total of 45 (37.5%) out of 120 bovine samples displayed a positive culture for *E. coli* (25 isolates from BM and 20 from NCD). In addition, 22 *E. coli* isolates recovered from chickens that died of AC, which were previously identified, were added to the collection for further analysis. Overall, 67 *E. coli* isolates were included in the present investigation.

### 3.2. Antimicrobial Susceptibility Testing and Screening for ESBL Production

The highest rates of antibiotic resistance in the 67 *E. coli* isolates were found for cefsulodine (67/67; 100%), followed by ceftazidime (56/67; 83.6%), streptomycin (55/67; 82.1%), cefotaxime (49/67; 73.1%), tetracycline (44/67; 65.7%), colistin (36/67; 53.7%), trimethoprim-sulfamethoxazole (29/67; 43.3%), nalidixic acid (27/67; 40.3%), ampicillin (24/67; 35.8%), enrofloxacin (20/67; 29.8%), chloramphenicol (19/67; 28.3%), cefoxitin (18/67; 26.8%), and meropenem and amoxicillin-clavulanic acid (17/67; 25.4%) for each. However, *E. coli* isolates exhibited lower frequencies of resistance to aztreonam (12/67; 17.9%), ceftiofur (8/67; 11.9%), gentamicin (5/67; 7.4%), ertapenem and imipenem (2/67; 3%) for each, and cefepime (1/67; 1.5%) (Table 2).

Avian isolates showed the most important antibiotic resistance rates for the majority of antibiotics tested except for cefotaxime, ceftazidime, and colistin, for which mastitis isolates displayed the highest resistance rates as shown in Table 2. Although diarrheal isolates showed the lowest resistance rates to the majority of antibiotics, they revealed higher resistance frequencies for ceftazidime, cefotaxime, cefoxitin, and gentamicin than those in avian isolates.

Of the 67 *E. coli* isolates, only 19 (28.3%) were ESBL producers. Among them, nine (47.3%) originated from chickens with colibacillosis, seven (36.8%) from cows with mastitis, and three (15.8%) from calves with diarrhea (Table 3). 

### 3.3. MIC of Colistin and Detection of Resistance Genes

A total of 36 colistin-resistant *E. coli* isolates were found (36/67; 53.7%) using the microdilution test with MICs of colistin ranging from 4 to 128 µg/µL (Table 3). Mastitis-associated isolates showed the greatest colistin resistance rate (18/25; 72%), followed by colibacillosis-related isolates (12/22; 54.5%), and finally calves’ isolates (6/20; 30%). The *mcr*-1 gene was detected only in 10 *E. coli* isolates, all collected from chickens with colibacillosis (10/36; 27.78%). However, the remaining *mcr* genes (2, 3, and 4) were not detected (Table 4). A statistically significant relationship was found between colistin resistance, carriage of the *mcr-*1 gene and the origin of isolates (*p* < 0.05) (Table 5).

### 3.4. Biofilm Formation Assay

A total of 49 of the 67 isolates (73.13%) formed biofilm (OD > 4 × 10^−3^), while 18 (26.87%) did not (OD ≤ 4 ×10^−3^). Among biofilm-forming isolates, 44 (65.67%) were classified as strong biofilm-forming isolates (OD > 16 × 10^−3^), while 5 strains (7.46%) were moderately biofilm-forming isolates (8 × 10^−3^ < DO ≤ 16 × 10^−3^). In this study, no strains were classified as weak biofilm formers (4 × 10^− 3^ < DO ≤ 8 × 10^−3^). The highest rate of biofilm-forming ability was observed in mastitis-associated isolates (20/25; 80%), followed by avian colibacillosis-associated isolates (17/22; 77.3%), and calve diarrheic isolates (12/20; 60%) (Table 3). Biofilm-forming ability in mastitis isolates was significantly higher than that in colibacillosis and diarrheic isolates (*p* < 0.05) (Table 5). Interestingly, the majority of colistin-resistant isolates were rather strong biofilm-forming (SBF) (26/36; 72.2%) or moderate biofilm-forming isolates (MBF) (2/36; 5.5%), whilst only nine colistin-resistant isolates were not biofilm producers (9/36; 25%) (Table 4). A statistically significant correlation was observed between colistin resistance and biofilm-forming ability (*p* < 0.05) (Table 5).

### 3.5. Detection Genes Encoding ESBL Enzymes and Other Resistance Markers

The *bla*_TEM_ gene encoding for ESBL production was found in 73.7% (14/19) of isolates (seven from cows, four from chickens, and three from calves). However, the *bla*_CTXM-g-1_ gene was only detected in nine (9/19; 47.3%) phenotypically ESBL-EC strains. All *bla*_CTXM-g-1-_carrying isolates were from chickens with colibacillosis, whilst none of the mastitis and diarrhea isolates carried this gene. The *bla*_SHV_ gene was detected in a single isolate of avian origin (1/19; 5.2%), whilst all isolates were free of the *bla*_CTX-M-g-8_ and *bla*_CTX-M-g-9_ genes (Table 4). A statistically significant relationship was found between ESBL production and the type of animal diseases (*p* < 0.05) (Table 5).

Among the 55 streptomycin-resistant isolates, *aad*A, *str*A, and *str*B genes were found in 23 (41.8%), 6 (10.9%), and 1 (1.8%) isolate, respectively. Of the 44 phenotypically tetracycline-resistant isolates, 15 (34.1%) were positive for the *tet*A gene (7 from cows, 5 from chickens, and 3 from calves). The *dfr*AI gene was detected in 11 out of the 29 (37.9%) trimethoprim-resistant isolates, whereas the *sul*1 gene was found in 7 isolates (7/29; 24.1%). Among the 19 chloramphenicol-resistant isolates, the *flo*R and *cmlA* genes were detected in 26.3% (5/19) and 5.2% (1/19) of isolates, respectively. All isolates were negative for *tet*B, *sul*2, and *dfr*IIV genes (Table 4).

### 3.6. Detection of Virulence and Biofilm Encoding Genes in CREC Isolates

The *fim*A gene was detected in 27 of the 36 CREC isolates (75%). Among these strains, 17 (63%) were from cows with mastitis and 9 (33.3%) from chickens with colibacillosis. In chickens, the *stx*1 and *stx*2 genes were observed in two isolates (9% for each), whilst isolates from mastitis and diarrhea were free of these genes. The *pap*C gene was observed in three and one isolates from mastitis and chickens, respectively. The *aer* gene encoding for aerobactin was detected in 7 out of the 22 chicken isolates (31.8%) and 5 out of the 25 mastitis isolates (20%). In addition, the *cnf*l gene was found in three isolates from both chickens and mastitis (13.6% and 12%, respectively). Mastitis isolates harboring VGs contained either the *fim*A gene alone or in combination with *aer*, *cnf*1, and *papC* genes. However, none of the diarrheal isolates was positive for the analyzed genes.

### 3.7. E. coli Phylogenetic Typing in CREC Isolates

Phylogroup analysis of the 36 CREC isolates showed that they belonged to three phylogroups: A (20/36; 55.5%), B2 (10/36; 27.7%), and D (6/36; 16.6%). Regarding subgroups, isolates were allotted as follows: A1 (20/36; 55.5%); D1 (4/36; 11.1%); B2_3_ (8/36; 22.2%); and B2_2_ and D2(2/36; 5.5% for each). While *E coli* isolates recovered from chickens were placed in five different subgroups (A1, D1, D2, B2_2_, and B2_3_), those originating from bovine mastitis and calves with diarrhea were allotted to four (A1, B2_3_, D1, and D2) and two subgroups (A1 and D1), respectively (Table 4). Interestingly, a statistically significant relationship between phylogroups and the type of animal diseases, biofilm-forming ability, and ESBL production was found (*p* < 0.05).

### 3.8. CREC Molecular Typing by ERIC-PCR

**Figure 1:**
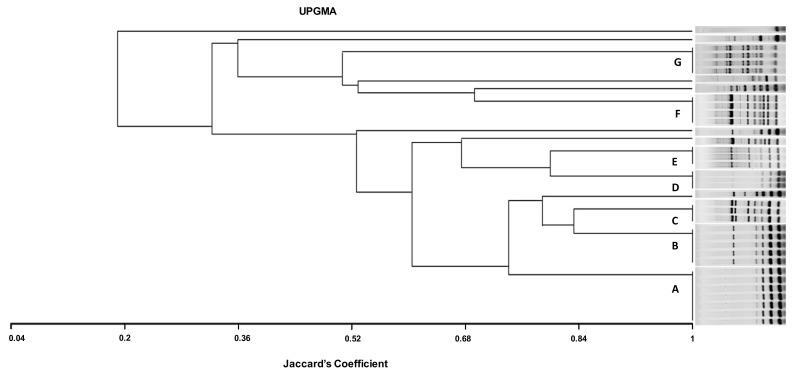

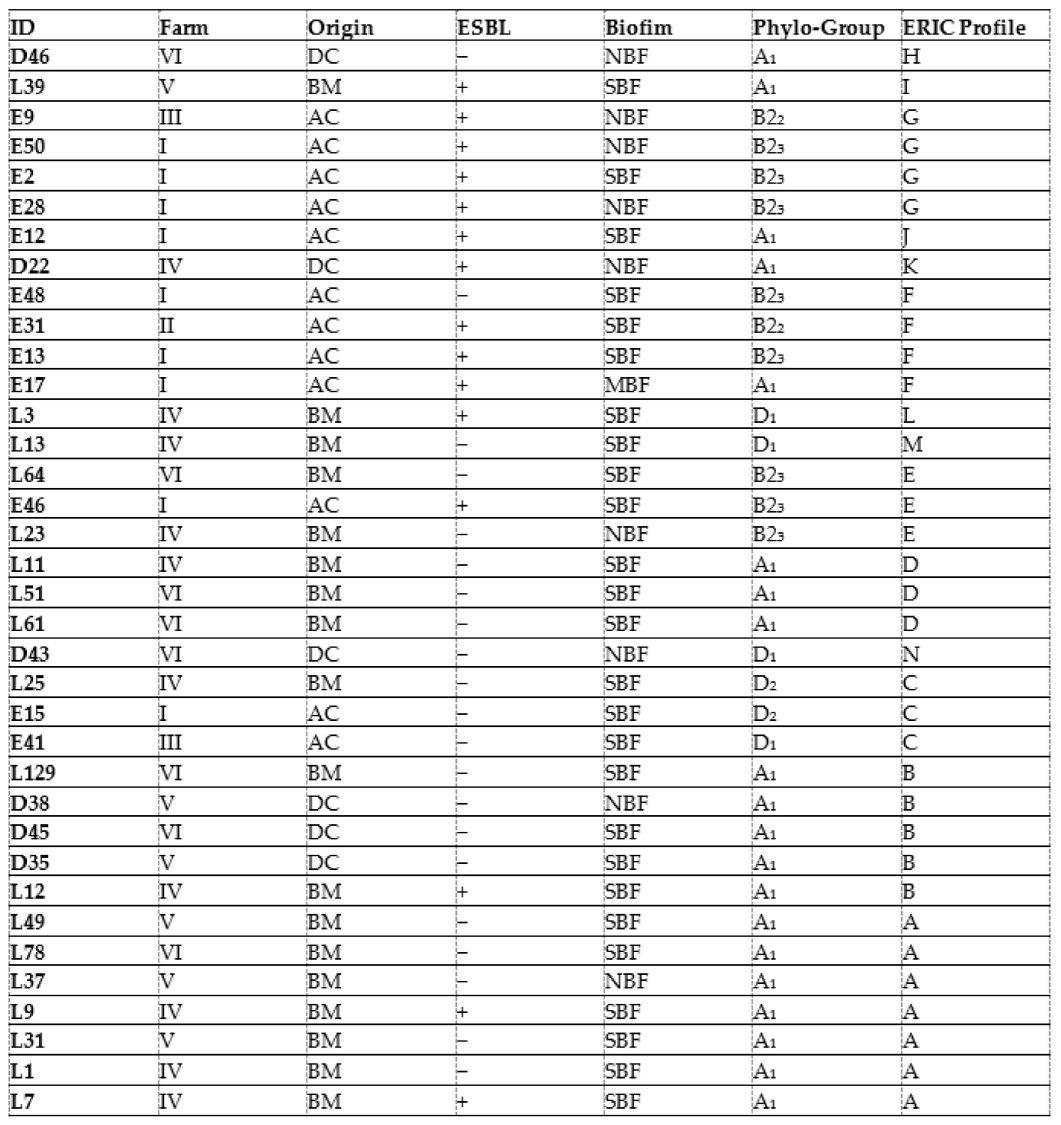
ERIC dendrogram representing the genetic relatedness and characteristics of 36 CREC isolates recovered from cows with mastitis, diarrheic calves, and chickens with colibacillosis in farms from Tunisia, using UPGMA (Unweighted Pair Group Method with Arithmetic mean using Jaccard’s coefficient). AC, avian colibacillosis; BM, bovine mastitis; DC, diarrheic calves; ESBL, extended-spectrum β-lactamases; A, B, C, D, F, and G, ERIC groups; NBF, non-biofilm forming; MBF, moderate biofilm-forming; SBF, strong biofilm forming.

Genetic relationship analysis for the 36 CREC isolates using ERIC-PCR showed 14 different ERIC types (ETs). Identical ETs were allocated letters from A to G, while unique ETs were assigned letters from H to N (Figure 1). The UPGMA method indicated seven different clones (A, B, C, D, E, F, G). The most prevalent ET was the type A (7/36; 19.4%) identified in the mastitis isolates, followed by the type B (5; 13.8%) found in isolates from diarrhea and mastitis, then the types F and G (4/36; 11.1%, for each) detected in avian isolates. The ETs C, D, and E (3/36; 8.3%, for each) were found in either mastitis or avian isolates. While ETs C and E were observed in mastitis and avian isolates, the type D was only detected in mastitis isolates (Table 4, Figure 1).

The dendrogram analysis indicates that the highest genetic diversity was observed in the eighteen CREC isolates collected from bovine mastitis with the presence of eight different ETs (A, B, C, D, E, M, L, and E). Among these ETs, the types A and D detected in mastitis isolates were found in different farms (FIV, FV, and FVI; FIV). Likewise, the ETs C and E including mastitis and avian isolates were found in three different farms, (FI, FIII, FIV) and (FI, FIV, FVI), respectively. Type B was detected in isolates collected from mastitis, diarrhea, and mastitis belonging to FIV, FV, and FVI. However, the ETs F and G included only avian isolates from three different farms (FI, FII, FII) (Table 4, Figure 1).

## 4. Discussion

In the present study, 67 *E. coli* isolates were recovered from bovine mastitis, calves’ diarrhea, and avian colibacillosis from different farms in Tunisia. Overall, isolates showed a high rate of multidrug resistance (83.6%). In cows, the highest frequencies of resistance were recorded for cefsulodine and ceftazidime (100% for each), cefotaxime (84%), streptomycin (80%), colistin (53.7%), and tetracyclines (40%). These frequencies were higher than those obtained in the study of Yu et al. (2020) [42], who recorded lower frequencies of resistance for cefotaxime, streptomycin, and tetracyclines in *E. coli* isolated from bovine mastitis (18.1%, 13.3%, and 12%, respectively). The resistance rate to enrofloxacin (4%) in our study was close to that reported in the study of Yu et al. (2020) [42] (4.8%). Contrarily, resistance rates to gentamicin (4%) in mastitis isolates were lower than those recorded in that study (12%) [42].

In calves, the same trend in resistance rates was observed regarding cefsulodine, ceftazidime (100% for each), and cefotaxime (85%). The lowest resistance rate was found for streptomycin (70%) and cefoxitin (75% for each), whilst the highest resistance rate to tetracyclines (75%) was recorded compared to mastitis isolates. These resistance rates are higher than those found by Srivani et al. (2017) [43].

In chickens, the highest rate of antibiotic resistance was found regarding cefsulodine (100%), streptomycin and nalidixic acid (95.4% for each), and tetracyclines (86.3%). The resistance rates for streptomycin (95.4%) and tetracyclines (86.3%) were higher than those found by Wang et al., 2021 [44] in chickens with colibacillosis in China. However, the resistance rate for gentamicin (4.5%) was lower compared to what was recorded in that study [44].

A total of 36 phenotypically colistin-resistant *E. coli* strains were found (36/67; 53.73%). The highest colistin resistance rate was detected in avian isolates (17/22, 77%) followed by bovine mastitis isolates (18/25, 72%) and diarrheal isolates (6/20, 30%). Although the microdilution test showed the highest rate of colistin resistance in mastitis isolates (18/36; 50%), the *mcr*-1 gene was detected in ten *E. coli* isolates all from chickens with colibacillosis (10/36; 27.78%). The colistin resistance rate found in avian isolates (27.78%) is close to that obtained in the study of Johar et al. (2021) [45] (28.5%) in chickens with colibacillosis from Qatar. In cows, the *mcr*-1 gene frequency was lower than that reported by Liu et al. (2020) [46], who detected 2% of colistin-resistant *mcr*-1 positive *E. coli* isolates collected from cows with mastitis. Conversely, the *mcr*-1 was not detected in any *E. coli* isolated from calves with diarrhea corroborating the results found by Umpiérrez et al., 2017 [47].

Interestingly, all *mcr-1*-positive *E. coli* isolates were multidrug-resistant, exhibiting resistance to common antimicrobials. This finding is in agreement with those found by Liu et al. (2020) [46].

In our study, *E. coli* isolates from the three different origins were free of the *mcr*-2, *mcr*-3, or *mcr*-4 genes. This result may be explained by the possession of other *mcr* gene variants or chromosomal mutation(s) [48]. Subsequently, further molecular investigations are needed to identify genes involved in colistin resistance in these isolates.

In the present investigation, a statistically significant association between colistin resistance, *mcr-*1 carriage, and biofilm formation ability was found supporting previous studies [49].

Strains isolated from chickens showed an important ESBL production rate (9/22, 40.90%) that was lower than that found by Parvin et al. (2020) (86%) [50] and higher than that observed in the study of Johar et al., 2021(3.8%) [45] in *E. coli* isolated from chickens. In cows with mastitis, 28% (7/25) of the *E. coli* isolates were phenotypically ESBL producers. This frequency is close to that obtained by Liu et al. (2020) [46], who recorded only 20% of ESBL-producing *E. coli* among 249 strains isolated from milk from cows with mastitis. The lowest rate of ESBL-producing isolates (3/20, 15%) was recorded in isolates from calves’ diarrhea.

The *bla*_CTXM-g-1_ was detected only in ESBL-producing avian isolates, whilst the *bla*_TEM_ gene was found in isolates from the three animal pathologies. This result is consistent with previous studies in which the *bla*_TEM_ gene was the most predominant ESBL encoding gene in *E. coli* isolated from avian colibacillosis, diarrheic calves, and bovine mastitis [20,51,52].

A total of 33 out of the 36 colistin-resistant *E. coli* isolates contained at least one of the following genes: *tet*A, *sul*1, *mcr*-1, *aad*A, *flo*R, *str*B, *dfr*AI *cml*A, *str*A, and *str*B, demonstrating the important antibiotic resistance pool in CREC isolates recovered from the three animal pathologies. Previous studies have demonstrated the detection of the same aforementioned genes in *E. coli* from cows with mastitis [42], avian colibacillosis [44], and diarrheic calves [51]. In the present study, antibiotics were widely used in farm animals either for treating mastitis, colibacillosis, and diarrhea or even to enhance their productivity. This practice promotes the dissemination of ARB that could reach food products, causing serious public health issues [53,54].

A total of 49 of the 67 isolates (49/67; 73.1%) formed biofilm. In cows, the percentage of biofilm-forming *E. coli* isolates (20/25, 80%) was lower than that found by [55], who reported 100% of biofilm-forming *E. coli* isolates from acute clinical environmental bovine mastitis in Brazil. In chickens, 77.3% (17/22) of isolates showed biofilm-forming ability. This frequency was markedly higher than that reported in the study of [49], in which only 45% of the APEC strains showed biofilm formation ability. The lowest rate (12/20, 60%) of biofilm formation was observed in calves when compared to the other origins. These results are higher than those reported in the study of [47], in which 45% of *E. coli* isolated from calves with diarrhea in Uruguay formed biofilm. Based on the observations of the biofilm formation assay, the study suggested that mastitis isolates were more biofilm producers than those of avian and calf origins. Our findings show that bovine mastitis, avian colibacillosis, and neonatal calf diarrhea may be biofilm-related diseases as biofilm-forming bacteria can be resilient to the immune system, antibiotics, and other treatments [17,49,56]. In addition, biofilm plays a key role in horizontal gene transfer (HGT) facilitated by highly dense cells nearby [57] which smooth the movement of RGs and virulence factors, especially under the selective pressure of antibiotics [58,59]. In the biofilm formation process, the key event is the attachment to the surface leading to subsequent aggregation and mature biofilm formation. This increases the stability of bacteria to cause diseases and enhances their drug resistance capacity [14].

In the present investigation, the most common virulence determinant found in *E. coli* isolates was the *fim*A gene (26/36, 72.2%) followed by *aer* (12/36, 33.3%), supporting anterior investigations [60]. The highest frequency of the *fim*A gene was found in mastitis isolates (17/25, 68%), corroborating previous findings by Jouini et al., (2021) [21] (66.67%). The *fim*A gene was found in 33.33% (9/27) of chicken isolates but was absent in diarrheal isolates. Genes encoding for Shiga toxins (*stx*1 and *stx*2) were detected only in avian isolates with lower rates (2/36; 5.5% for each) than those reported in the study of Elmonir et al. (2021) [61] (20% and 17.1% of isolates, respectively).

The co-occurrence of various VGs encoding for Shiga toxins known as diarrhea genic (*stx*1/2), aerobactin synthesis (*aer*), fimbria type I (*fim*A), and P-fimbriae involved in septicemia (*pap*C) emphasizes the fact that these avian isolates might be incriminated in the morbidity of chickens. Diagnosing APEC based on virulence genes is difficult since there is no specific set of virulence genes systemically associated with APEC [7]. However, based on the presence of specific genes, three isolates could be categorized as Shiga toxin-producing *E. coli* (STEC), containing *stx*1, *stx*2, or *stx*1 and *stx*2 genes [62]. STEC represents a public health threat if transmitted to humans as they can adhere to host epithelial cells and cause damage [63]. Furthermore, previous studies have provided evidence of potential zoonotic transmission of STEC isolates recovered from diarrheic cattle and their food products to humans, representing an emerging public health threat [61].

Mastitis isolates harbored *fim*A, *aer*, *cnf*1, *and pap*C. However, the presence of these genes could not determine whether these isolates are ExPEC or not. Other virulence genes associated with ExPEC such as *tra*T, *fyu*A, and *iut*A genes were found in *E. coli* from bovine mastitis in previous studies [10]. The *cnf*1 genes were detected in 24%of mastitis isolates, which is contradictory to the study of Suojala et al., 2011 [12] from Finland, in which all *E. coli* isolates from bovine mastitis were free of this gene. However, the *stx*1, *stx*2, and *hly*A genes were not detected in bovine isolates in our study following a previous study [12].

Although diarrheal isolates were free of the analyzed VGs, they might be reservoirs for other VGs such as *fim*H and *csg*A [44]. Animals and humans in contact with calves may become infected through their feces serving as reservoirs for antibiotic and virulence genes.

The results of the assessment of VGs showed high genetic heterogenicity among isolates as shown in other studies [47]. This heterogeneity might be the result of the acquisition and/or the deletion of genetic elements and localization of many virulence-associated genes on bacteriophages, plasmids, transposons, and pathogenicity islands contributing to either the gain or loss of these pathogenic attributes [64]. Although many VGs were not detected in *E. coli* isolates, the biofilm-forming ability might be due to the presence of pathogenicity islands and the expression of other virulence determinants.

It is important to take into account that given the small number of isolates, particularly those originating from calves, it is difficult to draw a clear conclusion about their virulence patterns. Thus, further molecular characterization of VRs in all isolates would be of great relevance to better elucidate the virulence background of *E. coli* incriminated in the three animal pathologies. Phylogroup distribution showed that most of the CREC isolates (20/36; 55.5%) were allotted to phylogroup A. In contrast to colibacillosis isolates that belonged mostly to the B2 phylogroup (8/12), mastitis and diarrheal isolates were mostly of the A phylogroup (13/18 and 5/6, respectively). This finding might be due to the difference in the origin of samples and the health status of animals. Indeed, avian *E. coli* isolates were recovered from dead chickens, whilst mastitis and diarrhea were isolated from diseased animals. The phylogroup results found in this study demonstrate the high pathogenicity of avian isolates compared to those from cows and calves. In previous studies, the virulent B2 group was frequently detected in ExPEC incriminated in severe human infections [12], which demonstrates the high zoonotic potential of avian isolates. Mastitis isolates were of phylogroups A1, D1, or B2. In contrast to phylogenetic type A, phylogroups D and B2 were considered virulent by Clermont et al. 2000 [38].

In this study, most of the mastitis isolates (72.2%) belonged to phylogroup A, a finding that agrees with similar studies that revealed the predominance of A and D phylogroups in *E. coli* isolates from cows with mastitis [65]. Contrarily, lower amounts of isolates were allotted to the D and B2 phylogroups, corroborating previous studies [65].

In the present investigation, the ERIC-PCR-based genotyping analysis of the 36 CREC isolates recovered from BM, NCD, and AC showed an important level of genetic diversity (14 ERIC profiles). The ERIC band patterns ranged from 1 to 10 bands with a size range from 100 to 2000 bp, comparable with reports from Egypt [61]. The twenty-two mastitis isolates showed the most identical ERIC profiles. Among these isolates, seven displayed the same ET (A), and three belonged to the ET (D), demonstrating clonal dissemination of *E. coli* among cows with mastitis. This result is consistent with that of Nüesch-Inderbinen et al., 2019 [10], who found high genetic diversity in *E. coli* isolates collected from cases of bovine mastitis. The six strains isolated from calves’ diarrhea showed four different profiles (groups B, H, K, and N); among them, ET (B) included three isolates. This result is in agreement with the study conducted by Gharieb et al. (2019) [66], who observed seven clusters in *E. coli* isolates recovered from diarrheic calves.

It is worth noting that the study of genetic relatedness using ERIC-PCR in avian isolates showed concordance with phylogenetic analysis results found in a previous study using the technique of pulsed-field electrophoresis (PFGE), considered as a reference technique for the molecular typing of bacteria [20]. In the present investigation, ERIC-PCR genotyping showed that some mastitis and colibacillosis isolates belonged to the same ET (groups C and E). This result is different from that of Grami et al. (2014) [67], who found no clonal relationship between strains from colibacillosis and bovine mastitis in Tunisia. In addition, ET (B) included strains from mastitis (*n* = 2) and calf diarrhea (*n* = 3), whilst no identical ETs were observed in chickens and calves’ isolates. ERIC-PCR analysis showed not only identical profiles but also unrelated patterns among CREC recovered from the three animal pathologies, which may reflect the diversity of CREC clones incriminated in these animal diseases in Tunisia.

The ETs (A) and (D) detected in mastitis isolates were found in different farms (FIV, FV, and FVI and FIV and FVI, respectively). This result indicates the dissemination of two different CREC clones in farms included in this study. This could be explained by the movement of animals between farms and their sharing of grazing and water sources. In addition, the (C) and (E) ETs including mastitis and avian isolates were found in three different farms (FI, FIII, and FIV and FI, FIV, and FVI, respectively). This finding demonstrates the involvement of the same CREC clones in avian colibacillosis and bovine mastitis in Tunisia. The ET (B) was detected in isolates collected from diarrhea and mastitis belonging to FIV, FV, and FVI, indicating that this clone disseminated among diseased calves and cows from different farms in Tunisia. However, the ETs (F) and (G) found in CREC of avian origin were circulating in three different farms (FI, FII, FII) but were absent in mastitis and calves’ isolates. There were no identical CREC clones between diarrheal and colibacillosis isolates. However, some diarrheal isolates (D22 and D43) showed close ETs with avian isolates clustered in the ETs (F) and (C), respectively.

ESBL and non-ESBL-producing CREC isolates from the three animal pathologies in the different farms were found to be related by ERIC genotyping. Moreover, ERIC-PCR has revealed a clonal relationship between *E. coli* biofilm-producer isolates from cows with BM following previous investigations in Brazil [68].

In the present study, the combinations of identical ETs, biofilm-forming ability, phylogenetic groups, virulence, and resistance profiles among some of the CREC isolates from the same or different animal pathology highlights the potential intra-species cross-transmission of these isolates and/or their genes in the study region.

On combining data, the majority of strong biofilm-producing CREC isolates were of either mastitis or colibacillosis origin and were allotted to the A, B2, and D phylogroups. These isolates displayed seven ETs that were circulating between cows, calves, and chickens, suggesting clonal dissemination of strong biofilm-producing CREC isolates with clinically relevant phylogroups in farms from Tunisia.

## 5. Conclusions

The present study showed a high prevalence of MDR *E. coli* (83.6%) isolated from BM, NCD, and AC. CREC and ESBL-EC isolates were shown from the three different origins. *E. coli* isolates harbored a combination of resistance, virulence, and β-lactamase-encoding genes and were assigned to the A, B, and D phylogroups. This is the first report of the biofilm formation ability in *E. coli* isolated from clinical cases of bovine mastitis, avian colibacillosis, and neonatal calves’ diarrhea in Tunisia. Our study revealed a high propensity of *E. coli* isolates recovered from diseased animals to produce biofilm, suggesting the importance of biofilm-forming ability in the pathogenesis process. Further, this paper sheds new light on the diversity and the clonality observed within CREC and ESBL-EC isolates from three different animal diseases in farms in Northern Tunisia.

## Figures and Tables

**Table 2:** Antibiotic resistance rates in 67 *E. coli* isolates recovered from bovine mastitis, calves’ diarrhea, and avian colibacillosis.

Antibiotic	Class	Mastitis (*n* = 25) *n* (%)	Colibacillosis (*n* = 22) *n* (%)	Diarrhea (*n* = 20) *n* (%)	Total (*n* = 67) *n* (%)
Ampicillin	β-lactams	8 (32)	10 (45.4)	6 (30)	24 (35.8)
Amoxicillin-clavulanic acid		2 (8)	10 (45.4)	5 (25)	17 (25.4)
Aztreonam		2 (8)	10 (45.4)	0 (0)	12 (17.9)
Cefotaxime		21 (84)	11 (50)	17 (85)	49 (73.1)
Cefoxitin		2 (8)	1 (4.5)	15 (75)	18 (26.8)
Ceftiofur		2 (8)	6 (27.3)	0 (0)	8 (11.9)
Cefsulodine		25 (100)	22 (100)	20 (100)	67 (100)
Ceftazidime		25 (100)	11 (50)	20 (100)	56 (83.6)
Cefepime		0 (0)	1 (4.5)	0 (0)	1 (1.5)
Imipenem	Carbapenems	1 (4)	1 (4.5)	0 (0)	2 (3)
Meropenem		4 (16)	12 (54.5)	1 (5)	17 (25.4)
Ertapenem		1 (4)	1 (4.5)	0 (0)	2 (3)
Tetracyclines	Tetracyclines	10 (40)	19 (86.3)	15 (75)	44 (65.7)
Enrofloxacin	Fluoroquinolones	1 (4)	16 (72.7)	3 (15)	20 (29.8)
Nalidixic acid	Quinolones	3 (12)	21 (95.4)	3 (15)	27 (40.3)
Gentamycin	Aminoglycosides	1 (4)	1 (4.5)	3 (15)	5 (7.4)
Streptomycin		20 (80)	21 (95.4)	14 (70)	55 (82.1)
Trimethoprim-sulfamethoxazole	Dihdrofolatreductase/Sulfonamide	6 (24)	15 (68.2)	8 (40)	29 (43.3)
Chloramphenicol	Phenicols	2 (8)	15 (68.2)	2 (10)	19 (28.3)
Colistin	Polymyxins	18 (72)	12 (54.5)	6 (30)	36 (53.7)

**Table 3:** Phenotypic resistance patterns, minimal inhibitory concentrations of colistin, ESBL production, and biofilm-forming ability in 67 *E. coli* isolates recovered from bovine mastitis, calves’ diarrhea, and avian colibacillosis in farms in Tunisia.

Farm	Strain ID	Origin	Phenotypic Resistance Profile	Colistin MIC (µg/mL)	Biofilm Formation
FI	E2 ^a^	AC	AMP/CFZ/CHL/ENR/MEM/NA/STR/SXT/TET/CST ^b^	8	SBF
	E3	AC	ATM/AMC/CAZ/CFZ/CTX/CHL/ENR/MEM/STR/XNL/NA/SXT	1	SBF
	E9 ^a^	AC	AMP/CFZ/CHL/ENR/MEM/NA/STR/SXT/TET/XNL/CST ^b^	1	NBF
	E12 ^a^	AC	AMP/CFZ/NA/STR/SXT/TET/CST ^b^	8	SBF
	E13 ^a^	AC	AMP/CAZ/CFZ/CTX/ENR/CST ^b^	8	SBF
	E15 ^a^	AC	ATM/AMP/AMC/CAZ/CFZ/CTX/CHL/ENR/ETP/MEM/NA/STR/SXT/TET/CST ^b^	4	SBF
	E17 ^a^	AC	ATM/AMP/AMC/CAZ/CFZ/CTX/CHL/ENR/GN/NA/STR/TET/XNL/CST ^b^	4	MBF
	E19	AC	ATM/AMC/CAZ/CFZ/CTX/CHL/ENR/NA/STR	0.25	SBF
	E22	AC	CFZ/ENR/NA/STR/SXT	0.5	SBF
	E28 ^a^	AC	AMP/CFZ/CHL/STR/TET/XNL/CST ^b^	4	NBF
	E35	AC	ATM/AMC/CAZ/CFZ/CTX/CHL/ENR/IMP/MEM/NA/STR/SXT/TET/XNL	0.5	SBF
	E42	AC	CFZ/CHL/ENR/MEM/STR/NA/SXT/TET	1	SBF
	E46	AC	AMP/CFZ/CHL/ENR/NA/STR/SXT/TET/CST ^b^	8	SBF
	E22	AC	CFZ/ENR/NA/STR/SXT	0.5	SBF
	E28 ^a^	AC	AMP/CFZ/CHL/STR/TET/XNL/CST ^b^	4	NBF
	E35	AC	ATM/AMC/CAZ/CFZ/CTX/CHL/ENR/IMP/MEM/NA/STR/SXT/TET/XNL	0.5	SBF
	E42	AC	CFZ/CHL/ENR/MEM/STR/NA/SXT/TET	1	SBF
	E46	AC	AMP/CFZ/CHL/ENR/NA/STR/SXT/TET/CST ^b^	8	SBF
	E47	AC	CFZ/MEM/NA/STR/TET	1	NBF
	E48	AC	ATM/AMC/CAZ/CFZ/CTX/NA/STR/TET/CST ^b^	4	SBF
	E50 ^a^	AC	ATM/AMP/AMC/CAZ/CFZ/CTX/ENR/MEM/NA/STR/SXT/TET/CST ^b^	4	NBF
FII	E18	AC	CFZ/CHL/ENR/NA/STR/SXT/TET	0.5	SBF
	E38	AC	CFZ/MEM/STR/CHL/NA/SXT/TET	0.5	NBF
	E31 ^a^	AC	CFZ/CHL/MEM/NA/STR/SXT/TET/CST ^b^	8	SBF
FIII	E20	AC	ATM/AMC/CAZ/CFZ/CTX/CHL/ENR/MEM/NA/STR/TET	0.5	SBF
	E27	AC	ATM/AMC/CAZ/CFZ/CTX/CHL/ENR/MEM/NA/STR/SXT/TET/XNL	0.25	SBF
	E41	AC	ATM/AMC/CAZ/CFZ/CTX/ENR/NA/SXT/TET/CST ^b^	4	SBF
FIV	L1	BM	CAZ/CFZ/STR/CST ^b^	32	SBF
	L3 ^a^	BM	AMP/CAZ/CFZ/CTX/FOX/STR/NA/CST ^b^	64	SBF
	L7 ^a^	BM	AMP/CAZ/CFZ/CTX/STR/CST ^b^	32	SBF
FIV	L9 ^a^	BM	AMP/CAZ/CFZ/STR/CST ^b^	64	SBF
	L11	BM	CAZ/CFZ/CTX/STR/CST ^b^	64	SBF
	L12 ^a^	BM	AMP/CAZ/CFZ/CTX/STR/CST ^b^	32	SBF
	L13	BM	CAZ/CFZ/STR/CST ^b^	128	SBF
	L19	BM	CAZ/CFZ/CTX	0.5	SBF
	L23	BM	AMC/CAZ/CFZ/CTX/STR/SXT/TET/CST ^b^	128	NBF
	L25	BM	CAZ/CFZ/CTX/STR/TET/CST ^b^	64	SBF
	L26 ^a^	BM	CAZ/CFZ/CTX	0.5	SBF
	D3	DC	CAZ/CFZ/FOX/TET	1	SBF
	D7	DC	CAZ/CFZ/CTX//FOX/MEM/TET	1	NBF
	D9	DC	CAZ/CFZ/CTX/TET	0.5	SBF
	D12	DC	CAZ/CFZ/CTX/STR/TET/ENR/CHL/GN/SXT	0.5	MBF
	D13	DC	AMC/CAZ/CFZ/CTX/FOX/NA/STR/SXT/TET	0.5	SBF
	D14	DC	AMC/CAZ/CFZ/ENR/FOX/NA/STR/SXT/TET	0.5	MBF
	D18	DC	AMC/CAZ/CFZ/FOX/STR/SXT/TET	1	NBF
	D22 ^a^	DC	AMP/AMC/CAZ/CFZ/CTX/CHL/ENR/GN/STR/SXT/TET/CST ^b^	32	NBF
FV	L31	BM	CAZ/CFZ/CTX/CST ^b^	64	SBF
	L37	BM	CAZ/CFZ/CTX/STR/TET/CST ^b^	32	NBF
	L39 ^a^	BM	CAZ/CFZ/CST ^b^	64	SBF
	L49	BM	CAZ/CFZ/CTX/GN/CST ^b^	32	SBF
	D24	DC	CAZ/CFZ/CTX/FOX/GN/STR/TET	1	SBF
	D29 ^a^	DC	AMP/CAZ/CFZ/CTX/TET	0.5	SBF
	D34	DC	CAZ/CFZ/CTX/FOX/SXT/TET	0.5	NBF
	D35	DC	AMP/CAZ/CFZ/CTX/FOX/STR/CST ^b^	32	SBF
	D38	DC	CAZ/CFZ/CTX/STR/SXT/TET/CST ^b^	32	NBF
	D41	DC	CAZ/CFZ/CTX/FOX/TET	0.25	MBF
	L58	BM	CAZ/CFZ/CTX/STR	1	NBF
	L61	BM	AMP/CAZ/CFZ/CTX/CHL/ENR/MEM/STR/SXT/TET/XNL/CST ^b^	128	SBF
	L62	BM	CAZ/CFZ/CTX//CHL/IMP/MEM/STR/SXT/TET	1	NBF
	L64	BM	AMP/CAZ/CFZ/CTX/MEM/STR/SXT/TET/CST ^b^	64	SBF
	L78	BM	ATM/AMP/AMC/CAZ/CFZ/CTX/ETP/MEM/STR/XNL/CST ^b^	128	SBF
	L79	BM	ATM/CAZ/CFZ/CTX/STR/SXT/TET	1	SBF
	L82 ^a^	BM	AMP/CAZ/CFZ/CTX/STR	0.25	SBF
	L94	BM	CAZ/CFZ/CTX/STR/TET	0.25	NBF
	L129	BM	CAZ/CFZ/CTX/STR/TET/CST ^b^	64	SBF
	D43	DC	AMC/CAZ/CFZ/CTX/SXT/STR/TET/FOX/CST ^b^	32	NBF
	D45	DC	AMP/CAZ/CFZ/CTX/FOX/STR/CST ^b^	64	SBF
	D46	DC	CAZ/CFZ/CTX/STR/CST ^b^	32	NBF
	D50	DC	CAZ/CFZ/CTX/FOX/STR/TET	1	SBF
	D52 ^a^	DC	AMP/CAZ/CFZ/CTX/FOX/STR	0.5	MBF
	D53	DC	CAZ/CFZ/CTX/CST ^b^	0.5	NBF

AMC, amoxicillin–clavulanic acid; AMP, Ampicillin; ATM, aztreonam; CAZ, ceftazidime; CFZ, cefsulodine; CHL, chloramphenicol; CTX, cefotaxime; ENR, Enrofloxacin; ETP, ertapenem; FOX, cefoxitin; GN, gentamycin; MEM, meropenem; NAL, nalidixic acid; STR, streptomycin; SXT, trimethoprim-sulfamethoxazole; TET, tetracyclines; XNL, ceftiofur; CST, colistin; AC, Avian colibacillosis; BM, bovine mastitis; DC, Diarrheic calves; ESBL: Extended-spectrum β-lactamase-producing strains. ^a^ ESBL producer, ^b^ Colistin-resistant strains (MIC ranging from 4 to 128 ug/mL), SBF: strong biofilm-forming, MBF: moderate biofilm-forming, NBF: non-biofilm-forming.

**Table 4:** Phenotypic and genotypic antibiotic resistance profiles, virulence genes, and biofilm-forming ability of 36 colistin-resistant *E. coli* isolates recovered from bovine mastitis, calves’ diarrhea, and avian colibacillosis in Tunisia.

Strain ID	Farm	Animal Pathology	Resistance Phenotypic Profile	Resistance Genes	Virulence Genes	Biofilm Formation	Phylo-Group	ERIC Profile
E12 ^a^	FI	AC	AMP/CFZ/NA/STR/SXT/TET/CST ^b^	*mcr*-1, *bla*_TEM_, *bla*_CTX-M-g-1_, *aad*A, *tet*A, *dfr*AI	*fim*A, *stx*1, *aer*	SBF	A_1_	J
E13 ^a^	FI	AC	AMP/CAZ/CFZ/CTX/ENR/CST ^b^	*mcr*-1, *bla*_CTX-M-g-1_, *flo*R	*fim*A, *stx*2	SBF	B2_3_	F
E15 ^a^	FI	AC	ATM/AMP/AMC/CAZ/CFZ/CTX/CHL/ENR/ETP/MEM/NA/STR/SXT/TET/CST ^b^	*mcr*-1, *bla*_CTX-M-g-1_, *tet*A, *aad*A, *flo*R, *str*A, *dfr*AI	*fim*A	SBF	D2	C
E17 ^a^	FI	AC	ATM/AMP/AMC/CAZ/CFZ/CTX/CHL/ENR/GN/NA/STR/TET/XNL/CST ^b^	*mcr*-1, *tet*A, *bla*_CTX-M-g-1_	---	MBF	A_1_	F
E2 ^a^	FI	AC	AMP/CFZ/CHL/ENR/MEM/NA/STR/SXT/TET/CST ^b^	*mcr*-1, *bla*_TEM_, *bla*_CTX-M-1_, *cmlA*	*fim*A, *stx*1, *stx*2, *aer*, *pap*C	SBF	B2_3_	G
E28 ^a^	FI	AC	AMP/CFZ/CHL/STR/TET/XNL/CST ^b^	*mcr*-1, *bla*_TEM_, *bla*_CTX-M-g-1_, *aad*A, *tet*A, *str*A	*fim*A, *aer*	NBF	B2_3_	G
E46 ^a^	FI	AC	AMP/CFZ/CHL/ENR/NA/STR/SXT/TET/CST ^b^	*mcr*-1, *bla*_CTX-M-1_	*fim*A, *aer*	SBF	B2_3_	E
E48	FI	AC	ATM/AMC/CAZ/CFZ/CTX/NA/STR/TET/CST ^b^	*dfr*AI, *aad*A	*aer*	SBF	B2_3_	F
E50 ^a^	FI	AC	ATM/AMP/AMC/CAZ/CFZ/CTX/ENR/MEM/NA/STR/SXT/TET/CST ^b^	*mcr*-1, *bla*_CTX-M-g-1_, *flo*R, *str*A, *dfr*AI	*fim*A, *aer*	NBF	B2_3_	G
E31 ^a^	FII	AC	CFZ/CHL/MEM/NA/STR/SXT/TET/CST ^b^	*mcr*-1	*fim*A, *aer*	SBF	B2_2_	F
E41	FIII	AC	ATM/AMC/CAZ/CFZ/CTX/ENR/NA/SXT/TET/CST ^b^	*tet*A	---	SBF	D_1_	C
E9 ^a^	FIII	AC	AMP/CFZ/CHL/ENR/MEM/NA/STR/SXT/TET/XNL/CST ^b^	*mcr-1*, *bla*_TEM_, *bla*_SHV_, *bla*_CTX-M-1_, *aad*A, *flo*R, *str*B, *dfr*AI	*fim*A	NBF	B2_2_	G
D22 ^a^	FIV	DC	AMP/AMC/CAZ/CFZ/CTX/CHL/ENR/GN/STR/SXT/TET/CST ^b^	*bla*_TEM_, *aad*A, *tet*A, *sul*1, *dfr*AI	---	NBF	A_1_	K
L1	FIV	BM	CAZ/CFZ/STR/CST ^b^	*aad*A	*fim*A, *cnf*1, *pap*C	SBF	A_1_	A
L7 ^a^	FIV	BM	AMP/CAZ/CFZ/CTX/STR/CST ^b^	*bla*_TEM_, *aad*A	*fim*A, *cnf*1, *pap*C	SBF	A_1_	A
L9 ^a^	FIV	BM	AMP/CAZ/CFZ/STR/CST ^b^	*bla*_TEM_, *aad*A	*fim*A, *cnf*1, *pap*C	SBF	A_1_	A
L12 ^a^	FIV	BM	AMP/CAZ/CFZ/CTX/STR/CST ^b^	*bla*_TEM_, *aad*A	*fim*A	SBF	A_1_	B
L25	FIV	BM	CAZ/CFZ/CTX/STR/TET/CST ^b^	*aad*A	*fim*A	SBF	D2	C
L11	FIV	BM	CAZ/CFZ/CTX/STR/CST ^b^	*aad*A, *tetA*	*fim*A, *aer*	SBF	A_1_	D
L13	FIV	BM	CAZ/CFZ/STR/CST ^b^	*aad*A	*fim*A, *aer*	SBF	D_1_	M
L23	FIV	BM	AMC/CAZ/CFZ/CTX/STR/SXT/TET/CST ^b^	*aad*A, *tet*A, *sul*1, *dfr*AI	*fim*A	NBF	B2_3_	E
L3 ^a^	FIV	BM	AMP/CAZ/CFZ/CTX/FOX/STR/NA/CST ^b^	*bla*_TEM_, *aad*A	*fim*A	SBF	D_1_	L
D35	FV	DC	AMP/CAZ/CFZ/CTX/FOX/STR/CST ^b^	*bla*_TEM_, *aad*A	---	SBF	A_1_	B
D38	FV	DC	CAZ/CFZ/CTX/STR/SXT/TET/CST ^b^	*aad*A, *tetA*, *sul*1, *dfr*AI	---	NBF	A_1_	B
L31	FV	BM	CAZ/CFZ/CTX/CST ^b^	---	*fim*A, *cnf*1	SBF	A_1_	A
L37	FV	BM	CAZ/CFZ/CTX/STR/TET/CST ^b^	*aad*A, *tet*A	--	NBF	A_1_	A
L49	FV	BM	CAZ/CFZ/CTX/GN/CST ^b^	---	*fim*A, *cnf*1	SBF	A_1_	A
L39 ^a^	FV	BM	CAZ/CFZ/CST ^b^	---	*fim*A	SBF	A_1_	I
D45	FVI	DC	AMP/CAZ/CFZ/CTX/FOX/STR/CST ^b^	*bla*_TEM_, *aad*A	---	SBF	A_1_	B
D43	FVI	DC	AMC/CAZ/CFZ/CTX/SXT/STR/TET/FOX/CST ^b^	*aad*A, *tetA*, *sul*1, *dfrAI*	---	NBF	D_1_	N
D46	FVI	DC	CAZ/CFZ/CTX/STR/CST ^b^	*aad*A	---	NBF	A_1_	H
L78	FVI	BM	ATM/AMP/AMC/CAZ/CFZ/CTX/ETP/MEM/STR/XNL/CST ^b^	*bla*_TEM_, *aadA*	*fim*A, *cnf*1	SBF	A_1_	A
L129	FVI	BM	CAZ/CFZ/CTX/STR/TET/CST ^b^	*aad*A, *tetA*	*fim*A	SBF	A_1_	B
L51	FVI	BM	CAZ/CFZ/CTX/STR/SXT/TET/CST ^b^	*str*A, *tet*A, *sul*1, *dfrAI*	*fim*A, *aer*	SBF	A_1_	D
L61	FVI	BM	AMP/CAZ/CFZ/CTX/CHL/ENR/MEM/STR/SXT/TET/XNL/CST ^b^	*bla*_TEM_, *strA*, *flo*R, *tetA*, *sul*1, *dfrAI*	*fim*A, *aer*	SBF	A_1_	D
L64	FVI	BM	AMP/CAZ/CFZ/CTX/MEM/STR/SXT/TET/CST ^b^	*bla*_TEM_, *strA*, *tet*A, *sul*1	*fim*A, *aer*	SBF	B2_3_	E

AMC, amoxicillin–clavulanic acid; AMP, Ampicillin; ATM, aztreonam; CAZ, ceftazidime; CFZ, cefsulodine; CHL, chloramphenicol; CTX, cefotaxime; ENR, Enrofloxacin; ETP, ertapenem; FOX, cefoxitin; GN, gentamycin; MEM, meropenem; NAL, nalidixic acid; STR, streptomycin; SXT, trimethoprim-sulfamethoxazole; TET, tetracyclines; XNL ceftiofur; CST, colistin; AC, Avian colibacillosis; BM, bovine mastitis; DC, Diarrheic calves; ^a^ Extended-spectrum β-lactamase-producing strains. ^b^ Colistin-resistant strains (MIC ranging from 4 to 128 ug/mL).

**Table 5:** Relationship between animal diseases and colistin resistance, *mcr*-1 gene carriage, ESBL production, and biofilm-forming ability of 67 *E. coli* isolates recovered from bovine mastitis, calves’ diarrhea, and avian colibacillosis in Tunisia.

	Mastitis (*n* = 25) *n* (%)	Colibacillosis (*n* = 22) *n* (%)	Diarrhea (*n* = 20) *n* (%)	Total (*n* = 67) *n* (%)	*p*-Value
Colistin resistance	18 (72)	12 (54.5)	6 (30)	36 (53.7)	0.000 *
*mcr*-1 gene	0 (0)	10 (27.7)	0 (0)	10 (14.9)	0.000 *
ESBL production	7 (28)	9 (40)	3 (15)	19 (28.3)	0.004 *
Biofilm formation	20 (80)	17 (77.3)	12 (60)	49 (73.1)	0.049 *

* Statistically significant relationship between animal diseases and colistin resistance, *mcr*-1 gene carriage, ESBL production, and biofilm-forming ability of *E. coli* isolates.

## Data Availability

The data presented in this study are available on request from Dr Ramzi Boubaker Elandoulsi.
